# Observation of Landau Level-Dependent Aharonov-Bohm-Like Oscillations in a Topological Insulator

**DOI:** 10.1186/s11671-020-03389-8

**Published:** 2020-08-26

**Authors:** Shiu-Ming Huang, Chien Lin, Sheng-Yu You, Pin-Cyuan Chen, Jai-Long Hong, Jyun-Fong Wong, You-Jhih Yan, Shih-Hsun Yu, Mitch M. C. Chou

**Affiliations:** 1 Department of Physics, National Sun Yat-Sen University, Kaohsiung, 80424 Taiwan; 2 Taiwan Consortium of Emergent Crystalline Materials, TCECM, National Sun Yat-Sen University, Kaohsiung, 80424 Taiwan; 3 Department of Materials and Optoelectronic Science, National Sun Yat-Sen University, Kaohsiung, 80424 Taiwan

**Keywords:** Aharonov-Bohm-like oscillations, Shubnikov-de Haas oscillation, Landau level

## Abstract

We study the quantum oscillations in the BiSbTe_3_ topological insulator. In addition to the Shubnikov-de Haas (SdH) oscillation, the Aharonov-Bohm-like (ABL) oscillations are also observed. The ABL oscillation period is constant at each Landau level (LL) which is determined from the SdH oscillation. The shorter ABL oscillation periods are observed at lower LLs. The oscillation period is proportional to the square root of the LL at temperatures. The ratio of the ABL oscillation period to the effective mass is weak LL dependence. The LL-dependent ABL oscillation might originate from the LL-dependent effective mass.

## Introduction

Aharonov-Bohm (AB) interference originates from the carrier wavefunction interference in a loop which might be patterned ring [1, 2], material geometric structure [3–6, 8–11], or carrier transport trajectory [12]. The magnetic field, *B*, through the loop will induce carrier wavefunction phase shift that leads to periodic wavefunction interference oscillations. This oscillation period is sensitive to the carrier transport characteristics, such as carrier coherence length and mobility [3, 12]. The quantum interference is an excellent tool to detect material transport characteristics and understand intrinsic mechanisms. Due to the short carrier coherence length and the small flux quantum, the quantum interference is mainly reported at high mobility nanowires or patterned nano-rings at low *B* [3–6, 8–11]. Reports on a macroscopic system at high *B* are rare. The works on AB quantum interference at high *B* are less investigated, and the related mechanism is less understood.

In this work, quantum oscillations were performed in a BiSbTe_3_ topological insulator macroflake at high *B*. In addition to the Shubnikov-de Haas (SdH) oscillation, the Aharonov-Bohm-like (ABL) oscillation was observed. The ABL oscillation period is *B*-dependent and is different from the traditional AB oscillation, which the oscillation period is independent of *B*. The observed ABL oscillation period is constant at each Landau level (LL), which is determined from the SdH oscillation. The shorter oscillation periods are observed at lower LLs. The oscillation period is proportional to the square root of the LL at temperatures. The ratio of the ABL oscillation period to the effective mass is weak LL dependence. The LL-dependent ABL oscillation might originate from the LL-dependent effective mass.

## Experimental Method

The growth condition of the BiSbTe_3_ single crystal is the same as our previous work on the topological insulators [13–16]. Our previous work demonstrated that TI with extremely high uniformity can be obtained using the RHFZ method [13–16]. Raman, EDS, and XPS spectrum proved that the crystal is BiSbTe_3_. The BiSbTe_3_ single crystal flakes were obtained using the Scotch-tape method. The cleaved flake geometry is roughly 3 mm in length, 2 mm in width, and 170 *μ*m in thickness. Magnetotransport measurements were performed using the standard six-probe technique in a commercial apparatus (Quantum Design PPMS) with a *B* of up to 14 T. The *B* was applied perpendicular to the large cleaved surface. The data points are taken per 100 Gauss at magnetic field region between 6 and 14 T in the steady magnetic field mode, instead of the sweeping magnetic field mode.

## Results and Discussion

**Fig. 1:**
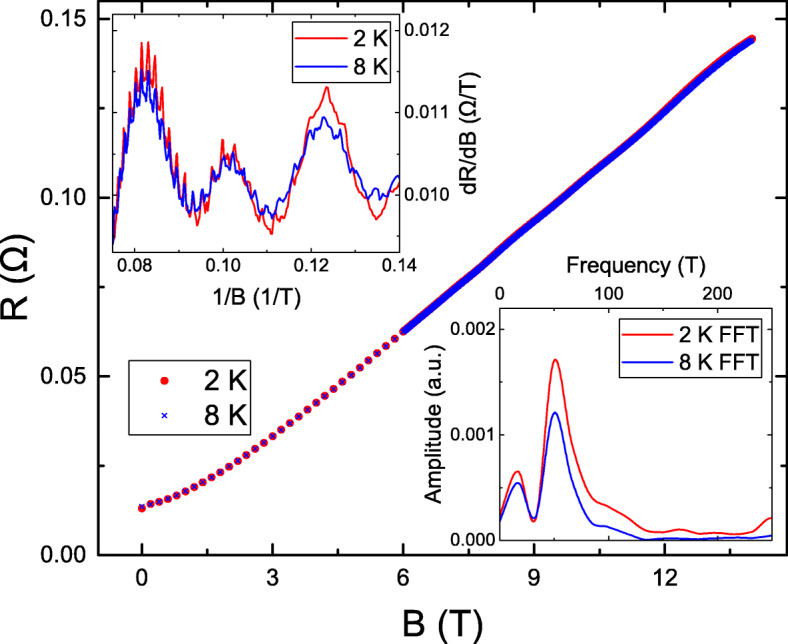
The magnetoresistance as a function of magnetic fields at 2 and 8 K. The top-left inset shows the *dR*/*dB* as a function of inverse magnetic fields. It reveal a periodic oscillations. The bottom-right inset shows the fast Fourier transform of the SdH oscillation and a sharp peak at 48 T for both 2 and 8 K

Figure 1 shows the magnetoresistances (MRs) as a function of *B*. The *R*(14T)/*R*(0T) reaches 10 and is higher than most reported values in Bi _*x*_*Sb*_2−*x*_*Te*_*y*_*Se*_3−*y*_ topological insulators [17–23, 23–33]. Both theoretical and experimental investigations support that the MR ratio is proportional to the carrier mobility [34], The measured high MR ratio supports the high quality of our BiSbTe_3_ sample. The top-left inset reveals the *dR*/*dB* as a function of 1/*B*. It reveals that periodic oscillations and oscillation peaks and dips are at the same *B* at 2 and 8 K. This is known as SdH oscillation that originates from a two-dimensional system. The SdH oscillation period corresponds to the Fermi momentum vector, *k*_*f*_. The bottom-right inset shows the fast Fourier transform (FFT) of the SdH oscillation. A sharp peak at 48 T is observed for both 2 and 8 K. Following the Onsager relation, one could estimate *k*_*f*_ through $F=\frac {\hbar k_{f}^{2}}{2e}$, where *F* is the SdH oscillation frequency. The *F*=48 T leads to the *k*_*f*_=3.8Å^−1^, which is consistent with the observed value from ARPES from a different batch of the same crystal and from reported values in literature [35]. That supports the high quality and uniformity of our BiSbTe_3_ crystal. As well as the SdH oscillation, the top-left inset reveals oscillations with a short period. To suppress the influence of the SdH oscillation and extract oscillation characteristics, the *d*^2^*R*/*dB*^2^ is performed.

**Fig. 2:**
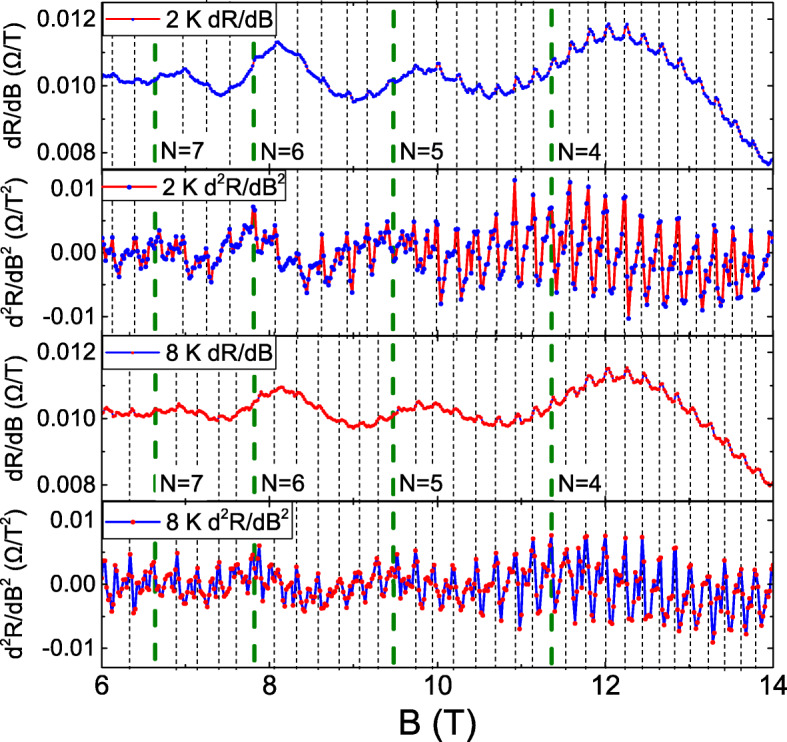
The *dR*/*dB* and *d*^2^*R*/*dB*^2^ as a function of *B* at 2 and 8 K. It shows periodic oscillations and the oscillation period is Landau level dependence

Figure 2 exhibits the *dR*/*dB* and *d*^2^*R*/*dB*^2^ as a function of *B* at 2 and 8 K. Dot lines label oscillation peaks in *d*^2^*R*/*dB*^2^, and long dash lines correspond to *B* of LLs that are determined from the extracted SdH oscillation frequency. The periodic oscillations is similar to the AB oscillation. The AB oscillation period is expressed as $\Delta B =\frac {\Phi }{A}$. *Φ* is the flux quantum, where $\frac {h}{e}$, and *A* is the geometry area looped by clock-count and anti-clock-count carrier trajectories in a confined structure. Due to the small flux quantum, the AB oscillation is mainly observed in confinement by artificial nanostructures [1, 2], such as nano-rings and nanowires [3–11]. Recently, it is reported that carrier elastic scattering trajectory might form a series of connected closed loops in a macroscopic system. A *B* flux through these loops would induce carrier wavefunction phase shift and lead to periodic ABL oscillations [12]. The extracted elastic scattering length is roughly 150 nm which corresponds to the oscillation period with 0.02 T and is consistent with our experimental observation.

**Fig. 3:**
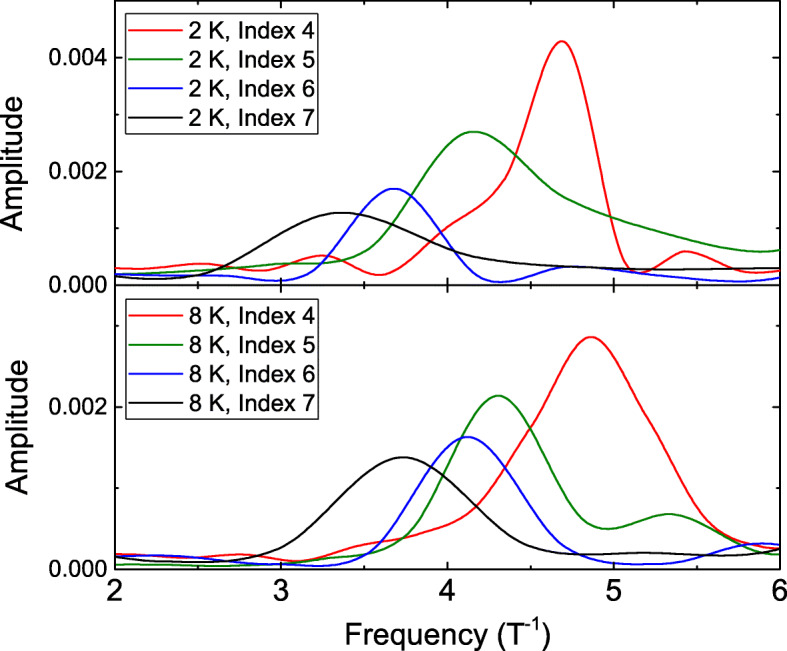
The fast Fourier transform of the *dR*/*dB* at different Landau levels and temperatures. The higher oscillation frequency peak is observed at lower Landau levels

Following the dot lines in Fig. 2, one could note that the oscillation period is constant at each LL and the oscillation period is shorter at lower LLs. This behavior is different from the traditional AB oscillation. To extract and determine these oscillation periods, FFT is performed at different LLs. Figure 3 shows the FFT at different LLs at 2 and 8 K, and it clearly reveals the higher oscillation frequency at lower LLs at 2 and 8 K.

A similar LL-dependent ABL oscillation is reported at the integer quantum Hall regime in semiconductor two-dimensional electron gas [36, 37]. It has been interpreted either as constructive interference of one-dimensional electron traveling along edge channels or as quantum wave interference of edge electrons. The carrier transport path in different edge channels leads to different effective areas in a confined pattern and eventually to different ABL oscillation periods in edge channels at different LLs [38–40]. Further studies on electric Fabry-Perot interferometers in integer and fractional quantum Hall regime reveal that the ABL oscillation period is related to the flux period by $\frac {\Phi }{f}$, where *f* is the fully occupied LL in the constrictions. The oscillation period is expected to be $\frac {\Phi }{A f}$, where *A* is the geometry area of the confined shape [41, 42].

Table 1 lists the extracted oscillation periods from the FFT at different LLs and temperatures. The analysis reveals that the ratio of the oscillation period to the square root of LL is constant at each temperature. This is different from the behavior of Fabry-Perot interferometer in which the oscillation is inversely proportional to LLs [41, 42]. On the other hand, the electric Fabry-Perot interference originates from carrier trajectory coupling between different LLs from inside and outside a confined pattern [37]. The oscillation is strongly related to the patterned geometry. There are no artificial patterns on the surface of our samples, and there should be no suitable coupling channels between different LLs. Furthermore, the geometry sizes of our samples are in the millimeter scale and the related AB oscillation period would be too small to be detected. Despite these differences from existing works, we think that aside from the geometric area and carrier coherence length, the intrinsic carrier characteristic might play a critical role on the LL-dependent ABL oscillation [3, 43].

**Table 1:** List of the extracted oscillation period at different Landau levels and temperatures

**Landau level**	**Oscillation period (*T*)**	${OP}/\sqrt {N}$	**OP/*m***_*cyc*_ (**T**/**m**_**0**_)
***N***	**OP**		
4 (2 K)	0.215	0.107	1.41
5 (2 K)	0.235	0.105	1.38
6 (2 K)	0.260	0.106	1.40
7 (2 K)	0.284	0.107	1.45
4 (8 K)	0.206	0.103	1.35
5 (8 K)	0.233	0.104	1.37
6 (8 K)	0.249	0.101	1.34
7 (8 K)	0.260	0.098	1.36

The oscillation period is proportional to the square root of Landau level

**Fig. 4:**
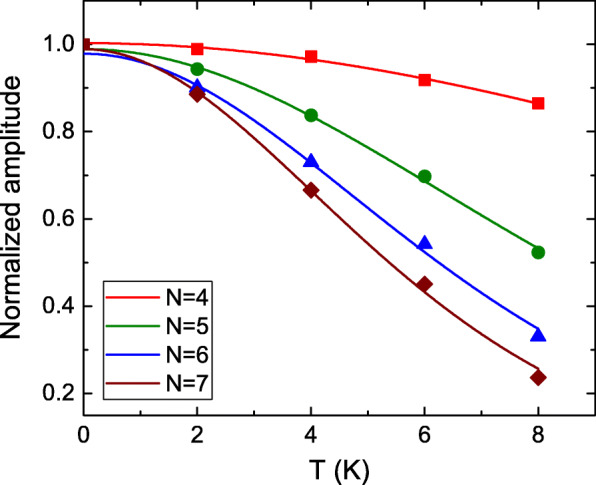
The extracted normalized SdH oscillation amplitude as a function of temperature at different Landau levels. It agrees well with the LK theory and reveals different tendency at different Landau levels

Following the Lifshitz-Kosevich (LK) theory, one can extract characteristic parameters of the transport carriers in the surface state of the topological insulator, and the temperature dependence of the amplitude of the SdH oscillation is expressed as

$$\Delta R_{xx}(T, B) \propto \frac{\lambda(T/B)}{\text{sinh}(\lambda(T/B))},$$

where $\lambda (T/B) = (2\pi ^{2}k_{B}Tm_{cyc})/(\hbar eB)$. Figure 4 shows the extracted normalized SdH oscillation amplitude as a function of temperature at different LLs. It agrees well with the LK theory and reveals different tendencies at different LLs. The fitting results support that the *m*_*cyc*_=0.152*m*_0_,0.170*m*_0_,0.185*m*_0_, and 0.191*m*_0_, where *m*_0_ is the free electron mass, for *N*=4, 5, 6 and 7, respectively. These values are consistent with the reported effective masses in topological insulators [21, 22]. This Landau level-dependent effective mass is recently observed in the 3D Dirac semimetal ZrTe_5_ [44]. However, the origin of the magnetic field-dependent effective mass is not clear yet. It needs further study to clarify the intrinsic mechanism. The different effective mass would directly deviate the intrinsic carrier transport characteristic at Fermi surface, such as Fermi velocity, which is directly related to the carrier phase coherence length. The higher effective mass would lead to lower coherence length that corresponds to the longer AB-like oscillation period. This is qualitatively consistent with our experimental observation. As shown in Table 1, the ratio of the AB-like oscillation period to the effective mass shows weak LL dependence. The Landau level-dependent effective mass might be one of the intrinsic effects that leads to the LL-dependent oscillation period.

**Fig. 5:**
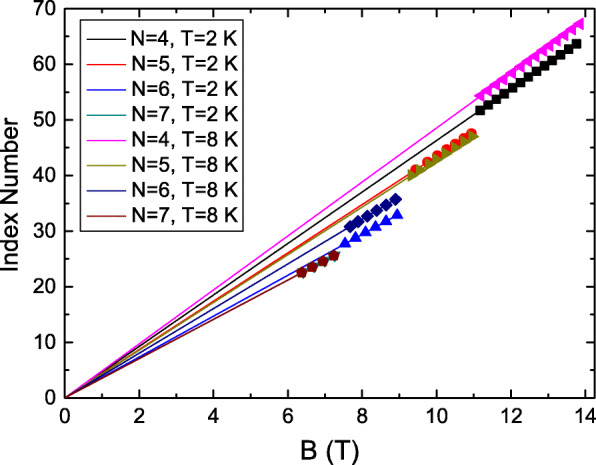
The AB oscillation index number as a function of *B* at different Landau levels and temperatures. The intercept is 0.25 which indicates a 0.5 phase shift in the plot of the AB oscillation. This supports the Berry phase is *π*

LL is a transport characteristic of a two-dimensional system. It indicates that the LL-dependent oscillation might have originated from the surface state carrier in TIs. Berry phase is a characteristic of transport carriers. Extracting the Berry phase might help identify the source of these LL-dependent periodic AB oscillations. We define the AB oscillation index number by dividing the corresponding *B* of oscillation peaks in *dB*/*dB* by the related oscillation period in the LL. It reveals that the index number of oscillation peaks in *dB*/*dB* corresponds to *N*+0.25, where *N* is integer, for all oscillations in different LLs and temperatures. This further supports that the AB oscillation period is related to LLs. Figure 5 shows that AB oscillation index numbers are proportional to *B* at different LLs and temperatures. The intercept is 0.25 which indicates a 0.5 phase shift in the plot of the AB oscillation. This supports the Berry phase is *π* and the observed AB oscillations might be the carrier transport characteristic of the surface state in our BiSbTe_3_ topological insulator [45].

## Conclusion

We have reported the quantum oscillations in a BiSbTe_3_ topological insulator macroflake. In addition to the Shubnikov-de Haas (SdH) oscillation, it reveals Aharonov-Bohm-like (ABL) oscillation. The ABL oscillation period is *B*-dependent. The ABL oscillation period is constant at each Landau level (LL). The shorter oscillation periods were observed at lower LLs, which was determined through the SdH oscillation. The oscillation period is proportional to the square root of the LL at different temperatures. The ratio of the ABL oscillation period to the effective mass is weak LL dependence. The LL-dependent ABL oscillation might originate from the LL-dependent effective mass.

## Data Availability

The datasets generated during and/or analyzed during the current study are available from the corresponding authors on reasonable request.

## Abbreviations

EDS: Energy-dispersive X-ray spectroscopy
XPS: X-ray photoelectron spectroscopy
ARPES: Angle resolved photoemission spectroscopy
SdH: Shubnikov-de Haas
